# Supplementary material for: TDLAS second harmonic demodulation based on Hilbert transform
Source: PLoS One. 2022 Dec 5;17(12):e0278724. doi: 10.1371/journal.pone.0278724 (PMC9721476; doi:10.1371/journal.pone.0278724)
Supplement: Attachment — Submitted filename: Response to Journal Requirements and Reviewers.docx [file pone.0278724.s001.docx]

We would like to thank all reviewers for the positive ratings. We appreciate all positive reviews that recognized our novelty and merit.

Response to the Journal Requirements

1) Please ensure that your manuscript meets PLOS ONE's style requirements, including those for file naming. The PLOS ONE style templates can be found at <https://journals.plos.org/plosone/s/file?id=wjVg/PLOSOne_formatting_sample_main_body.pdf> and

<https://journals.plos.org/plosone/s/file?id=ba62/PLOSOne_formatting_sample_title_authors_affiliations.pdf>

Response: Manuscript has been revised according to the requirements.

2) We note that the grant information you provided in the ‘Funding Information’ and ‘Financial Disclosure’ sections do not match.

When you resubmit, please ensure that you provide the correct grant numbers for the awards you received for your study in the ‘Funding Information’ section.

Response: The correct grant numbers for the awards we received for our study is provided.

Funder: Wuhan National Laboratory for Optoelectronics

Award Number: 2019WNLOKF011

Grant Recipient: Guohua Kang, Doctor's degree

Response to Reviewer #1

1) In the Experiment section, the optical path is filled with gas in a Coke bottle, and second harmonic is gotten with two methods that of LIA and Hilbert transform. By comparing the error of the two harmonics obtained by the two methods, the Hilbert transform method is validated. However, just as the paper said, the experiment is only conducted under one concentration condition of 15%. Therefore, the conclusion is not convincing enough. Please Supply the experiments and results under other concentration conditions.

Response: We agree with your comment that further experiments under other concentration conditions will help to reinforce our conclusions. Your suggestion provides a direction for our next research. Unfortunately, the research project has ended, and the experimental equipment we made has been delivered. At present, we do not have sufficient conditions to support further supplementary experiments.

2) When comparing the difference of the two methods, only the error of harmonic is analysed. To describe the full performance of the method, some other performance evaluations should be validated, such as algorithm speed, stability and so on.

Response: In terms of the speed of the algorithm, for the same data, the processing time of the proposed algorithm is about 5 times that of the lock-in amplification method, and the main processing time is spent on filtering and calculating the Hilbert transform of the signal; Compared with lock-in amplification method, the proposed method is more sensitive to noise, so band-pass filtering is used in this paper to improve the stability of the algorithm against noise.

3) Please supply some experimental details, like the cutoff frequency of band-pass filtering.

Response: Our data is processed on a computer. The modulation frequency *f* in our experiment was 21000 Hertz, so in the program the cutoff frequency of the first band-pass filtering was set as [0.5**f*, 2.5**f*], which is [10500 Hz, 52500 Hz], and the cutoff frequency of the second band-pass filtering was set as [0.8**f*, 1.2**f*], which is [16800 Hz, 25200 Hz]. A detailed description of experimental including the cutoff frequency of band-pass filtering has been added to the revised manuscript. See line 278-281 of the revised manuscript.

4) Please supply a physical picture of the experiment.

Response:

In the experiment, the gas in a bottle containing a little coke was detected. It has been corrected in the revised manuscript.

Original text (Line 250): In the experiment, this section of the optical path is filled with gas in a Coke bottle.

Modified text (Line 272): In the experiment, the gas in a bottle containing a little coke was detected.

Response to Reviewer #2

1) The author declared "The concentration of CO2 in the atmosphere is close to 400 ppm" in Line 189. It is not true for ambient CO2 concentration being much higher.

Response: The statement here is not rigorous enough. It has been corrected in the revised manuscript.

Original text (Line 189): The concentration of CO_2_ in the atmosphere is close to 400 ppm.

Modified text (Line 190): Global average atmospheric carbon dioxide is about 400 ppm.

2) A clear explanation for every panel of Fig. 2 is necessary.

Response: A clear explanation for every panel of Fig. 2 has been included in the revised manuscript.

(Line 202-214): The transmitted light signal in Fig. 2(a) consists of low-frequency sawtooth wave and high-frequency harmonics (the figure shows the signal for only half of the scanning period), which are the common results of the intensity modulation of injection current and nonlinear absorption; In Fig. 2(b), all signals except the first harmonic (1*f*) and second harmonic (2*f*) are filtered out, corresponding to Eq. (8); Fig. 2(c) is the envelope of the signal in Fig. 2(b). Since the intensity modulation leads to a strong gas-independent RAM signal in the first harmonic, the envelope change in the figure is not obvious, the gas related signal can be seen by zooming on the vertical axis and looks like a lying “S” plus some high-frequency component. These high-frequency component is the second harmonic signal that is transformed to 1*f*, corresponding to Eq. (16); Then the 1*f* component of |z1(t)| is obtained by band-pass filtering and presented in Fig. 2(d).

3) An explanation or discussion for the "error increases as the laser scans away from the center of the spectral line" is necessary.

Response: An explanation for the "error increases as the laser scans away from the center of the spectral line" has been included in the revised manuscript.

(Line 230-239): What stands out in Fig. 4 (b) is that the relative error increases gradually as the laser scans away from the center of the spectral line. This is due to the brick-wall bandpass filtering used in this simulation, which introduces a ringing artifacts error between the two signals. Fig. 5 shows the error between the second harmonic demodulated by the two methods.

**Fig. 5. The error between the second harmonic demodulated by the two methods.**

Therefore, the relative error increases gradually on both sides of the spectrum where the amplitude of the second harmonic gradually approaches zero.

4) A detailed experiment setup is necessary, such as the product model of digital acquisition, lock-in amplifier used in the manuscript, and their parameters setting.

Response: Our raw data were collected using an oscilloscope with a sampling frequency of 25MHz, and all the processing of the raw data described in this paper was carried out on a computer, so the lock-in amplifier was implemented by programming. The following figure shows our experimental setup.

The circuit board is a lock-in amplifier designed by ourselves, but the data from this lock-in amplifier is not used in this paper.

A detailed experiment setup such as the product model of digital acquisition has been included in the manuscript.

(Line 274-276): The TDLAS signal is detected by an InGaAs photodiode (LSIPD26-1) and collected by a digital oscilloscope (Rigol DS1102Z-E) with a sampling frequency of 25 MHz.

Response to Reviewer #3

1) A demodulation method for tunable diode laser absorption spectroscopy (TDLAS) second harmonic based on the Hilbert transform is proposed in this paper. But, Hilbert transform is a classical method and has been used to obtain an analytic representation in the field of gas detection. So, it is recommended that authors should not shy away from the current Hilbert transform methods, and proposed an improved Hilbert transform to process the gas data with better performance. Similar references are as following:

1. Efficient terahertz absorption gas sensor with Gaussian process regression in time- and frequency-domain, Sensors and Actuators B: Chemical, 2022.

2. Computational Doppler-limited dual-comb spectroscopy with a free-running all-fiber laser, APL Photonics, 2019.

Response: Hilbert transform is a classical method and has been used to obtain an analytic representation in the field of gas detection. But these two articles you recommend have little relevance to the topic of this paper. To the best of the authors' knowledge, the second harmonic demodulation method proposed in this paper has not been proposed in the previous literature. However, our scope of knowledge is limited and if you know of any other papers that have proposed a similar approach, you are welcome to recommend them to us.
