# Supplementary material for: TDLAS second harmonic demodulation based on Hilbert transform
Source: PLoS One. 2022 Dec 5;17(12):e0278724. doi: 10.1371/journal.pone.0278724 (PMC9721476; doi:10.1371/journal.pone.0278724)
Supplement: Attachment — Submitted filename: Response to Reviewers.docx [file pone.0278724.s002.docx]

We would like to thank all reviewers for the positive ratings. We appreciate all positive reviews that recognized our novelty and merit.

Response to Reviewer #1

1) Why only one band-pass filter is used in the introduction of Hilbert transform method in FIG. 1, while two band-pass filters are used in the experiment, which are inconsistent with each other?

Response: The third step “1*f* component extraction” in FIG 1 is actually implemented by bandpass filtering as well. We mentioned this in line 155 and 156 of the revised manuscript of the last edition. And considering that “1*f* component extraction” can better express the practical significance of this step than simply calling it “bandpass filtering”, this paper refers to this step as “1*f* component extraction”. The revised manuscript has made a supplementary explanation for this.

2) In this paper, the second harmonic signal obtained by phase-locked amplification method is taken as the real signal, and compared with the second harmonic signal obtained by Hilbert transform method, so as to show the relative error of Hilbert transform method. However, the second harmonic obtained by the phase-locked amplification method is also different according to its different filter parameters. At this time, the error of the signal will change when the two methods are compared. Then, is the phase-locked amplification method used in this paper the optimal effect of demodulation of the second harmonic (that is, are the relevant parameters of the phase-locked amplification process best)? If not, then the error analysis will lose some credibility. Please elaborate on this question.

Response: The lock-in amplifier was implemented by programming. We used orthogonal vector lock-in amplifier to avoid distortion caused by inaccurate phase of reference signal, and therefore the main factors affecting its performance are reference signal frequency and filter parameters.

The frequency of the reference signal was set as 42000 Hz, which is twice the frequency of modulation frequency (that is, equal to the frequency of the second harmonic). And in order to speed up the filtering process, the inverse Fourier transform is used in the programming script to implement a brick-wall low-pass filtering. When setting the filtering cutoff frequency, we ensured that the second harmonic signal can be restored as much as possible. The following two figure shows the amplitude spectrum of the pre-filtered and post-filtered signals in two orthogonal vector “sin” and “cos”,

Fig. 1. The amplitude spectrum of the pre-filtered and post-filtered signal in “sin” vector.

Fig. 2. The amplitude spectrum of the pre-filtered and post-filtered signal in “cos” vector.

We found that the energy of the second harmonic line mainly concentrated in the frequency below 200 Hz, and there was also a small amount of energy between 200-500Hz. Therefore, we set the cut-off frequency as 1000 Hz to avoid the distortion of the second harmonic waveform caused by filtering as much as possible, and also filtered out the high-frequency noise.

3) Is it more convincing to take the stability of the second harmonic signal obtained by the Hilbert transform method and the ability to characterize the concentration of the gas to be measured as the evaluation basis of the performance index of this method instead of just comparing with the phase-locked amplification method?

Response: The main purpose of this paper is the demodulation of the second harmonic, and the ability to characterize gas concentrations is a secondary application of this method. In the case of low absorbance, the Hilbert transform method can be used to demodulate the second harmonic signal. Therefore, the second harmonic obtained by the proposed method is compared with that of the lock-in amplifier method.

The stability of the proposed algorithm mainly depends on the SNR of the second harmonic signal. In the case of low SNR, the passband bandwidth of bandpass filtering should be set narrower.

4) In line 286 to 287, in order to express the similar results obtained by the two methods, describing specific performance indicators may be better than "almost overlap".

Response: The statement here has been revised in the manuscript.

Original text (Line 286-287): Similar to the simulation results, the signals demodulated by the two methods are almost overlap.

Modified text (Line 290-293): The results are similar to those of simulation and the signals demodulated by the two methods are almost overlap. Except near the local minimum of the second harmonic, the relative error of other positions is less than 2.5%, and the relative error at the center of the second harmonic is 0.6 ~ 0.7%.

5) The scanning mode in this paper is triangular wave scanning. It can be seen from Figure 8b that the second harmonic signal corresponding to the rising part of triangular wave and the signal corresponding to the falling part seem to show different error characteristics. Is it better to switch to a sawtooth scan?

Response: The scanning mode is not the main factor affecting the error, but switching to a sawtooth scan does provide better signal consistency (the waveform is basically the same from every scan).
